# The genomic diversity of Salmonella Enteritidis from poultry and human clinical cases in Israel

**DOI:** 10.1099/mgen.0.001878

**Published:** 2026-10-27

**Authors:** Janet Perry, Katya Arnold, Anat Wiseman, Yael Markus, Ido From, Eugenia Yakunin, Assaf Rokney, Ehud Elnekave

**Affiliations:** 1 The Koret School of Veterinary Medicine, Robert H. Smith Faculty of Agriculture, Food and Environment, Hebrew University of Jerusalem, Jerusalem, Israel; 2 Poultry Health Laboratories, Egg and Poultry Board, Kiryat Malachi, Israel; 3 Public Health Laboratories - Jerusalem, Ministry of Health, Jerusalem, Israel

**Keywords:** antimicrobial resistance, poultry, public health, *Salmonellosis*, zoonosis

## Abstract

*Salmonella* Enteritidis, a leading cause of foodborne illness worldwide, is often linked to poultry. Despite the limited genetic variation of the serotype, distinct lineages have been shown to circulate at both national and global scales, and invasive and antimicrobial-resistant strains have emerged. In Israel, despite active surveillance and mitigation measures in the poultry sector, the pathogen persists in commercial layer hen flocks and continues to cause human clinical infections. We aimed to characterize the genetic diversity and antimicrobial resistance (AMR) determinants of *S*. Enteritidis isolates from poultry and human clinical cases in Israel and to compare these findings with the genetic diversity of this serotype found globally. Whole-genome sequencing was performed on *S*. Enteritidis isolates from surveillance samples collected from layer hen flocks (*n*=48) and human clinical cases (*n*=29) between 2019 and 2022 and compared to a representative global dataset of sequences (*n*=115). We identified a genetically diverse *S*. Enteritidis population in Israel. A dominant clade I and a smaller clade IV were ST11 and closely related to each of the major ‘Atlantic’ and ‘Global’ lineages circulating globally. These clades demonstrated shared circulation between poultry and human populations which may suggest poultry-associated transmission. In addition, three smaller distinct Israeli clades were not part of the global clades. These included clades II and III of ST11 and clade V of ST183, a rarely described sequence type in poultry. Mutations in the *gyrA* gene (D87G, D87Y and S83F) conferring resistance to fluoroquinolones were present in both human (6.9%) and poultry (10.4%) isolates from Israel, highlighting the importance of continued antimicrobial resistance surveillance in this serotype. These findings establish a genomic baseline to support control and mitigation policy for *S*. Enteritidis in Israel and contribute to the understanding of its genomic diversity globally.

**Impact Statement**

*Salmonella* Enteritidis remains a persistent public health challenge despite longstanding surveillance and control efforts in the poultry sector. In Israel, it is the second most frequently detected serotype in human clinical cases. It is seldom detected in poultry breeding flocks yet persists in layer flocks at low levels. This study provides the first whole-genome sequencing (WGS)-based characterization of *S*. Enteritidis from both poultry and human clinical populations in Israel and their comparison to globally circulating *S*. Enteritidis strains.

We provide genomic evidence of high similarity between *S*. Enteritidis isolates collected from poultry and humans, giving support to the ongoing efforts for targeted control in the poultry sector. However, the detection of ST183, a sequence type previously associated with wildlife and rarely reported in poultry, emphasizes the potential importance of additional non-poultry reservoirs.

By integrating national isolate data with a representative global dataset, we situate the majority of Israeli *S*. Enteritidis population within the broader international genomic landscape. Yet several distinct clades exist, revealing local genetic diversity.

The detection of fluoroquinolone resistance due to mutations in a target gene in both human and poultry isolates adds to growing international evidence of resistance in this serotype and highlights the risk that treatment options for invasive disease may be compromised.

Together, these findings illustrate the resolution that WGS brings to *S*. Enteritidis epidemiology and point to the importance of sustained poultry sector control, antimicrobial resistance surveillance and broader monitoring beyond poultry hosts.

## Data Summary

The sequence data of the *Salmonella* Enteritidis isolates newly sequenced for this project (*n*=77) have been deposited in the National Center for Biotechnology Information (NCBI) under BioProject PRJNA1465885, with accession numbers SRR38584797 through SRR38584873. All previously published metadata and accession numbers downloaded from the NCBI and used in this project can be found in Table S1. Metadata and accession numbers can be found in Table S2.

## Introduction

Non-typhoidal *Salmonella* (NTS) is one of the leading causes of foodborne illness worldwide and a major public health concern [1]. Infection with NTS in humans usually presents as self-limiting gastroenteritis; however, infants, the elderly and immunocompromised individuals are at increased risk of invasive disease which may require treatment and hospitalization. Of the over 2,600 serotypes of *Salmonell*a *enterica*, *S*. Enteritidis continues to be the most frequently reported in the European Union [2] and the USA [3].

Zoonotic infections caused by *S*. Enteritidis are most commonly associated with poultry, particularly chicken; however, it is a generalist serotype and has the capacity to be found in a broad range of food-producing hosts, including pigs, cattle and other poultry such as turkeys [2]. The association with chickens is likely to be enhanced by the ability of the bacteria to colonize the large intestine and the ovary and infect the developing egg *in vivo* before the shell has formed [4]. Thus, transmission to humans can be via consumption of infected eggs or faecal contamination of egg or meat products. This also means that chicks can be infected pre-hatching, enabling vertical transmission from breeding stock to the commercial laying hens or broilers.

The emergence of *S*. Enteritidis as a dominant serotype in poultry occurred in the second half of last century, likely filling an ecological niche that opened following the eradication of serotypes Gallinarum and Pullorum from commercial poultry [5]. Subsequently, a pandemic of human *S*. Enteritidis infections associated with poultry products emerged in the Americas and Europe in the 1980s and worldwide in the 1990s [6]. Recently, this global dissemination was linked to the industrialization and globalization of poultry production and specifically to the international trade of contaminated breeding stock [7]. Historically, *S*. Enteritidis has not been associated with high levels of antimicrobial resistance (AMR) [8]; however, recent reports describe increasing resistance to clinically important antimicrobials such as fluoroquinolones [8–10], as well as the emergence of multidrug-resistant strains [11]. Exacerbating the threat to public health is the involvement of this serotype in many large-scale *Salmonella* outbreaks [12–15]. In addition, in sub-Saharan Africa, strains of *S*. Enteritidis that cause invasive disease with a high fatality rate have emerged [16], and high levels of invasive disease have also been reported in China [11].

In Israel, *S*. Enteritidis continues to keep its place as a dominant serotype affecting human health. In 2022, it was the second most frequently detected serotype, after *S*. Muenchen/Virginia, accounting for 933 of 3,171 (29.4%) of *Salmonella* isolates from human samples [17].

The Israeli poultry industry comprises two main sectors: table eggs (layer) and meat (broiler) production. Both sectors have a pyramidal structure with a small number of breeder flocks supplying a much larger number of commercial flocks. Breeder stock is imported in both sectors (parent stock for the layers and grandparent stock for the broilers) predominantly from Europe and North America [personal communication: Wiseman, A; Egg and Poultry Board (EPB)]. The majority of layer farms are privately owned and run and are not integrated, whereas the broiler farms are vertically integrated. The layer sector produces ~2.2 billion table eggs annually for domestic supply, supplemented with 100–200 million imported eggs, predominantly from Europe, during holiday periods when consumption increases. Domestic broiler chicken and turkey production (~260 million and 9 million birds annually, respectively) supplies 100% of local demand [18].

Dedicated control and mitigation actions in the poultry sector, including vaccination of breeder stock and culling of entire flocks upon detection, are carried out in accordance with international standards. Despite almost complete eradication from the breeder flocks and a marked decline since 2003 when mandatory vaccination against *S*. Enteritidis was introduced in commercial egg-laying flocks [19], *S*. Enteritidis has maintained its presence in the latter with an annual incidence of 1.0–2.4% between 2018 and 2023 [20].

*S.* Enteritidis presents limited genetic variation [21, 22]. Globally, the population is predominantly represented by a singular sequence type (ST)11 [23]. Multiple national and international studies have applied whole-genome sequencing (WGS) to determine the genetic variation and epidemiological trends occurring within *S*. Enteritidis [7, 9–11, 24–26]. Two major lineages circulating globally were first described in the human population [16] and were later identified also in poultry and referred to as the Global and Atlantic clades [7]. In addition, a study from the USA demonstrated that many NTS serotypes, including *S*. Enteritidis, could be divided into genetically distinct subpopulations that vary in their association with different host species [21]. Recently, to better detect within-serotype variation, several frameworks including multilevel genome typing (MGT) have been developed [27]. This WGS-based hierarchical framework identifies an increasing number of loci that generate STs at nine different levels. At each level, STs are grouped into clonal complexes (CCs) based on allele similarity. This enables comparison of isolates at multiple resolutions with MGT4CC shown to be useful in identifying lineages present in a population at a national level [28].

Given the economic and public health importance of *S*. Enteritidis in Israel, routine sample collection of *S*. Enteritidis occurs as part of active surveillance in the poultry sector and passive surveillance of human clinical disease in the public health sector; however, sequencing of samples is limited, and knowledge of its genetic diversity is lacking. We aimed to use WGS on isolates from poultry and human clinical cases in order to:

- Characterize the genetic diversity, including the presence of AMR determinants in *S*. Enteritidis in Israel.
- Compare the genetic diversity and lineages found in Israel to those found globally.

## Methods

### Study populations

Analysis A: The Israeli Veterinary Services (IVS) and the EPB carry out an active *Salmonella* surveillance programme across all layer hen farms and breeding flocks in Israel [29]. The surveillance aligns with the European Commission Regulation No. 517/2011 [30], with the modification of using environmental drag swabs for sampling. Samples are processed at the EPB *Salmonella* Laboratory in accordance with the International Organization for Standardization protocol ISO-6579-1 for detection of *Salmonella*. Between January 2019 and December 2022, (1) an average of 1,906 commercial layer flocks (range: 1,767–2124) were tested annually. Detection rates of *S*. Enteritidis ranged from 1.4 to 2.4% of flocks, with a total of 143 flocks testing positive over the 4 year period; (2) an average of 28 (range: 25–31) layer breeder flocks were tested annually, with none testing positive for *S*. Enteritidis; and (3) an average of 260 (range: 230–289) broiler breeder flocks were tested annually, of which a single flock tested positive for *S*. Enteritidis (not included in this analysis). In addition, there was a single incidental finding of *S*. Enteritidis in a commercial broiler flock of young chicks (<2 weeks old) which was included in this analysis.

Overall, 145 *S*. Enteritidis isolates collected from poultry were available. These originated from 124 farms from 38 localities (defined as an individual town, village or agricultural settlement). Fifteen farms were the source of more than one positive flock over the study period. We purposively selected 50/145 (34.5%) isolates for WGS to represent the national geographic distribution, with 1 to 3 isolates selected from 35 of the 38 localities, with no farm represented more than once. More than one isolate was selected from localities with a higher burden of positive flocks, and in all except one case, these isolates from the same locality were chosen from different collection years. Selection was balanced across the study period with 13 isolates collected in 2019, 13 from 2020, 14 from 2021 and 10 from 2022.

Human clinical isolates of *S*. Enteritidis (*n*=30) collected between January 2019 and December 2022 were selected from the frozen (−80 °C) isolate collection of the National Reference Center for *Salmonella* of the Ministry of Health (MOH), to which all confirmed clinical *Salmonella* isolates from sentinel and regional laboratories are submitted and a selection that represents geographic and demographic distribution as well as the variety of sample sources is stored. Associated patient data was not disclosed, yet isolate selection by the MOH collaborators was conducted using a stratified random approach to represent a broad geographic distribution of patients, balanced across the study period with 7 isolates from 2019, 8 from 2020, 7 from 2021 and 8 from 2022.

The number of isolates (of both poultry and human origin) selected for sequencing in this study was limited given budgetary constraints. Using standard binomial probability, a sample size of at least 29 isolates was calculated to provide a ≥95% probability of detecting at least one isolate of a major lineage (defined as at least 10% of the circulating *S*. Enteritidis strains).

Analysis B: In addition to *S*. Enteritidis isolates collected in Israel (analysis A), we identified *S*. Enteritidis strains from published databases of two studies to represent the genetic variation found globally. From the study of Elnekave *et al*. [21], demonstrating the genetic variation found in the USA, we chose a selection of isolates (*n*=34) representing five subpopulations (subpopulations 1–4 plus an ‘ungrouped’ subpopulation), comprising human (*n*=21), poultry (*n*=11) and ‘other’ hosts (*n*=2). To further represent global genetic variation and capture historical diversity, we selected isolates (*n*=81) from the dataset of Li *et al*. [7]. We selected isolates from human (*n*=28), poultry (*n*=32) and ‘other’ hosts (*n*=21), representing all continents. Isolates were included where host, collection location, collection year and Illumina short-read sequences were available; sequences were downloaded from the National Center for Biotechnology Information (NCBI) (https://www.ncbi.nlm.nih.gov/pathogens/) (Table S1, available in the online Supplementary Material).

### DNA extraction and WGS

Israeli isolates were brought on Luria–Bertani agar plates to the Laboratory of Veterinary Public Health at the Koret School of Veterinary Medicine, Rehovot. DNA was extracted using the DNeasy Blood and Tissue kit (QIAGEN, Venlo, Netherlands) or Mag-Bind Bacterial DNA Kit (Omega, GA, USA) and quantified with a NanoDrop 2000 Spectrophotometer (Thermo Fisher Scientific, USA). Samples were sent to the Center for Genomic Technologies at the Hebrew University (Jerusalem, Israel) or to SeqCenter (Pittsburgh, PA, USA) for sequencing. In both centres, library preparation was performed using the tagmentation-based Illumina DNA Prep kit (https://www.illumina.com/products/by-type/sequencing-kits/library-prep-kits/illumina-dna-prep.html). Paired-end reads of 150 bp were generated using the NextSeq platform (Illumina, San Diego, CA).

For construction of a reference genome, one representative isolate from our poultry cohort (Agri_104) was selected for hybrid assembly. In addition to the Illumina short-read sequencing, DNA was extracted from this isolate using the Blood and Cell Culture DNA Midi kit (QIAGEN, Venlo, Netherlands) and sent to SeqCenter for long-read sequencing on the Oxford Nanopore platform (Oxford, UK), generating 81,029 reads with a mean read length of 6,340 bp. A hybrid assembly of 4,720,429 bp was created with Unicycler v0.5.0 [31] and visualized with Bandage v0.8.1 [32].

### Genetic analysis

#### Reference-based alignment for construction of phylogenetic trees

Illumina raw reads were trimmed and checked for quality control using FastP v0.23.4 [33]. Snippy v4.6.0 [34] with default settings was used to align both the Israeli sequences (analysis A) and the Israeli and global sequences (analysis B) to the reference genome. Phage regions within the reference genome were found using PHASTER [35, 36] and masked using a previously described script [37]. Recombination regions were detected and masked using Gubbins v3.3.5 [38]. The core SNP alignment for the Israeli sequences (analysis A) consisted of 3,586 SNP sites (pairwise SNP range: 0–1,658; median: 182.5) and 20,360 SNP sites (pairwise SNP range: 0–14,153; median: 309) for the alignment with global isolates (analysis B). Sequences from ten Israeli isolates that were identical (no SNP pairwise difference) to sequences from other (Israeli) isolates in the dataset were removed for the purpose of reconstructing the phylogenetic trees. Maximum-likelihood trees for each analysis were reconstructed using RAxML v8.2.12 [39] and rooted to a *S*. Dublin outgroup strain (accession number SRR1636692). Branch support for the trees was assessed using 1,000 bootstrap replicates. The trees were visualized using FigTree v1.4.4 [40], packages ggtree v3.6.2 [41], treeio v1.22.0 [42], tidytree v0.4.2 [43] and tidyverse v2.0.0 [44] in R v4.2.0 [45] and the Interactive Tree of Life (iTOL) v7.4.2 [46].

### *De novo* assembly for genomic characterization

*De novo* genome assemblies of the Israeli isolates were conducted using ‘SPAdes’ *de novo* assembler v3.12.0 [47] with the ‘careful’ option to reduce short indels and minimize the number of mismatches in the final assembly. Assembly quality was evaluated using QUAST v5.2.0 [48]; assemblies with an N50 below 30,000 nt (*n*=1) or a genome size larger than expected (>8 Mbp) (*n*=2) were excluded from all analyses. Genome indexing was performed using Bowtie 2 v2.4.5 [49, 50] and SAMtools v1.16.1 [51]; average coverage depth per assembly was calculated using BBMap v39.06. All included sequences had an average coverage depth greater than 40. Contigs were used as input for *in silico* serotyping using the *Salmonella In Silico* Typing Resource (SISTR) platform v1.1.1 [52], and multilocus sequence typing was performed using Multilocus Sequence Type (MLST) v2.23.0 (https://github.com/tseemann/mlst) with the PubMLST database [53]. AMR genes and point mutations were detected using AMRFinderPlus v3.12.8 with database v2024-01-31.1 [54] run locally with default detection thresholds (minimum 50% coverage and 90% identity). Plasmid replicons were identified and typed using PlasmidFinder v2.1.6 [55] (database updated 7 March 2024), run locally with default detection settings (60% coverage and 90% identity).

### Identification of genetic subpopulations

Israeli clades were defined based on the major branching observed in the maximum-likelihood tree, all with high bootstrap support (≥80%). We cross-checked the Israeli clades against clustering inferred by the hierarchical Bayesian Analysis of Population Structure algorithm using RhierBAPS [56] on the core genome alignment, at two hierarchical levels. Subpopulation structure of both Israeli and global isolates was additionally assessed with MGT; for this purpose, paired-end Illumina reads were uploaded to the online platform [57] and MGT4 clonal complex (CC) assignments were identified.

### Phenotypic antimicrobial susceptibility

AMR was assessed using ComASP Colistin (Liofilchem, Teramo, Italy, 2025) for colistin resistance, and Sensititre susceptibility plates suitable for non-fastidious Gram-negative isolates (plate CMV4AGNF, Thermo Scientific, West Sussex, UK, 2019) for a panel of 14 antimicrobials (i.e. amoxicillin–clavulanic acid, ampicillin, azithromycin, cefoxitin, ceftriaxone, chloramphenicol, ciprofloxacin, gentamicin, meropenem, nalidixic acid, streptomycin, sulfamethoxazole–trimethoprim, sulfisoxazole and tetracycline). Colistin and Sensititre microdilution plates were manually inspected and susceptibility interpretation of tested antimicrobials was according to the Clinical and Laboratory Standards Institute (CLSI) cutoffs for *Salmonella* strains described in the guidelines [58]. Azithromycin has no CLSI breakpoint; therefore, the National Antimicrobial Resistance Monitoring System (NARMS) cutoffs were used [59]. Human cutoffs were used for the data to be relevant to the public health risk.

### Data summary and statistical analysis

Data wrangling and analysis were conducted using R v4.2.0 [45]. Location of samples was mapped using ArcGIS Pro 3.2.2 (ESRI, Redlands, CA, USA). Metadata and accession numbers from sequenced isolates and those downloaded from the NCBI database can be found in Tables S1 and S2.

## Results

### Genetic diversity of *S*. Enteritidis in layer hens and humans in Israel (analysis A)

A total of 77 isolates, 48 from poultry from a wide geographic distribution (Fig. 1a) and 29 from humans, were included in the study of Israeli isolates. Of the poultry isolates, 47 were from layer hen flocks, and 1 isolate was detected in a broiler flock.

**Fig. 1.**
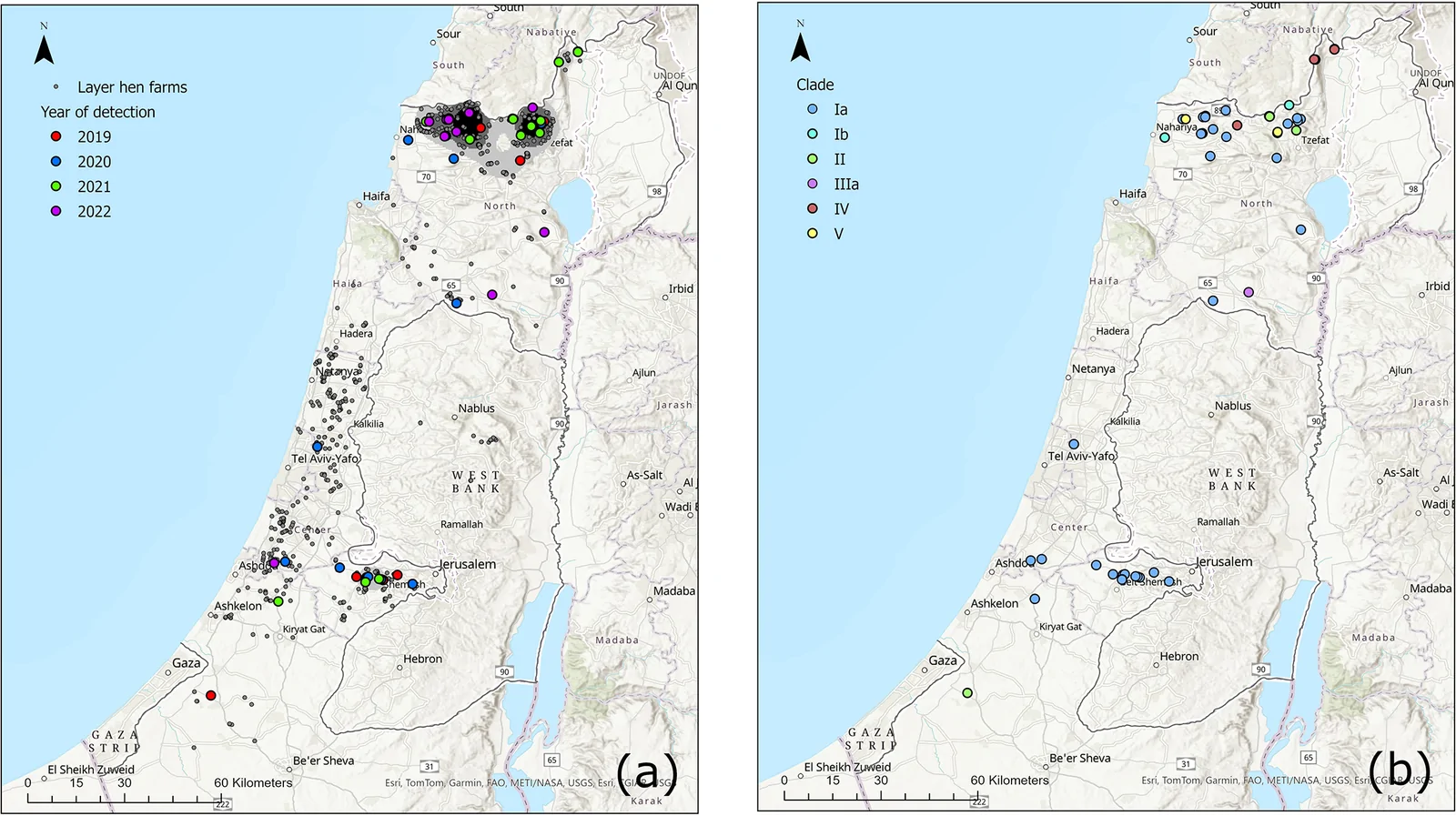
Temporal and geographical distribution of *S*. Enteritidis isolates detected in poultry layer flocks in Israel and included in this study. (**a**) Location of all layer hen farms (grey markers), with areas of increasing farm density indicated by the grey-scale kernel (grey gradient). Isolates are coloured according to year of detection. (**b**) Isolates are coloured according to phylogenetic clade.

### Genetic diversity

Overall, five clades were identified (Fig. 2). These clades were generally aligned with MGT4CC and hierBAPS assignment (Table S3). Clades I–IV were of ST11 (*n*=73, 94.8%) with four isolates classified as ST183, forming clade V.

**Fig. 2.**
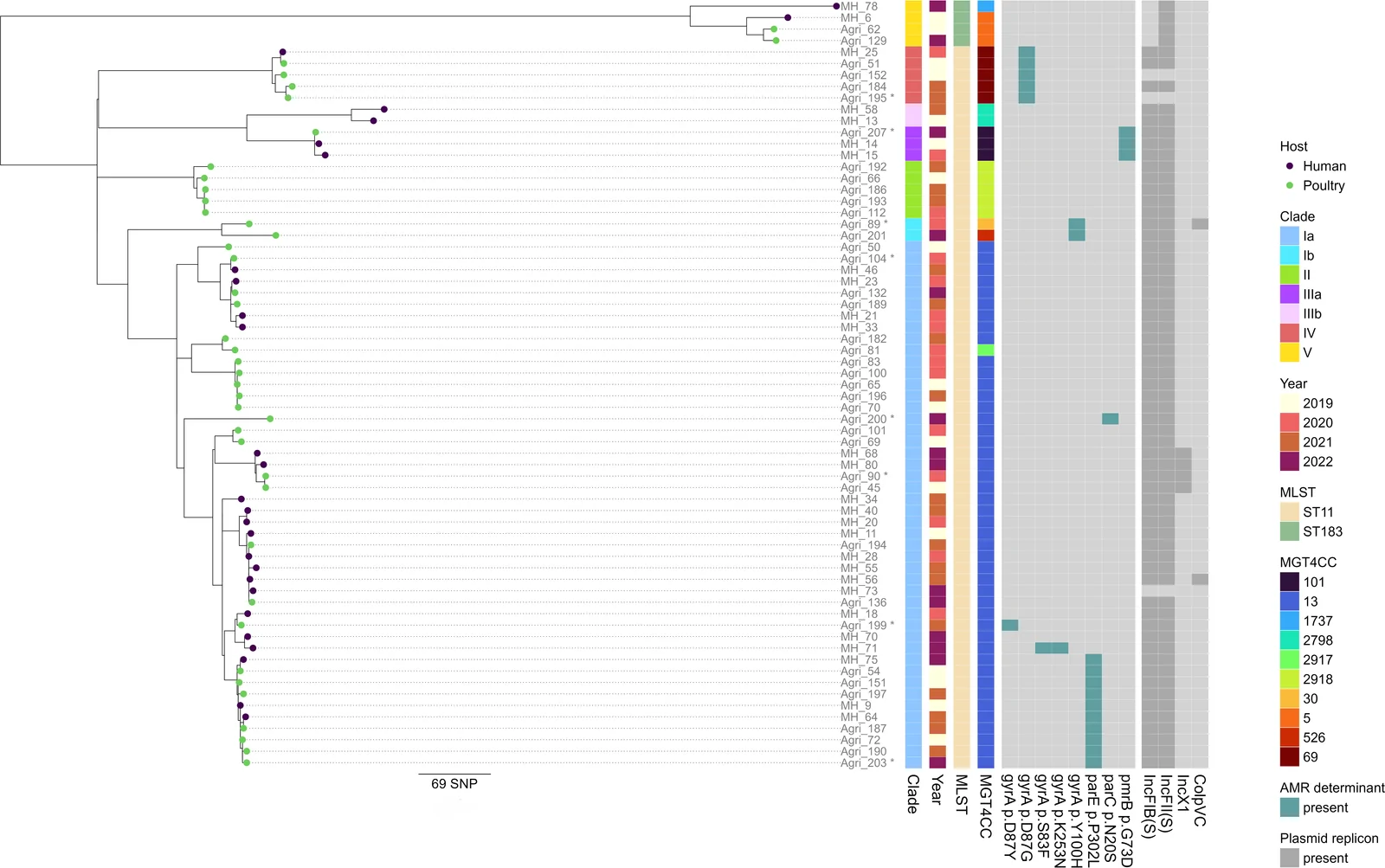
A maximum-likelihood phylogenetic tree reconstructed using non-duplicate WGS sequences of *S*. Enteritidis isolates collected from layer flocks (*n*=39) and humans (*n*=28) in Israel between January 2019 and December 2022. A hybrid assembly of *S*. Enteritidis (Agri_104) strain was used as a reference genome. A *Salmonella* Dublin strain (SRR1636692) was used to root the tree (not shown). Branch support for the trees was assessed using 1,000 bootstrap replicates. Defined clades were supported with bootstrap values of >80. Tip colours indicate host. Asterisks (*) indicate isolates in which antimicrobial susceptibility was tested *in vitro* (see results in Tables 1 and S4). Data shown in the heatmap includes clade, year of sampling, MLST, subpopulation (MGT4CC), chromosomal point mutations and presence of plasmid replicons.

Of the ST11 isolates, clade I was dominant and consisted of both human and poultry isolates. This clade was divided into two subclades: Ia and Ib. Subclade Ia contained the majority of both poultry (34/48, 70.8%) and human isolates (22/29, 75.9%). Isolates in this clade represented all collection years and were broadly distributed across the country (Fig. 1b). Ten sequences in Ia were duplicates: two human isolates and one poultry isolate were indistinguishable from another human isolate, while the remaining seven were poultry isolates indistinguishable from other poultry isolates. Pairwise differences in clade I ranged from 0 to 377 SNPs [median=152; interquartile range (IQR)=59–168].

Subclade Ib and clades II–IV were small (*n*≤5 isolates), mainly found in the northern regions of the country. Clade II was a homogeneous, poultry-only clade (median pairwise SNP difference=22.5, IQR=7.75–42), free of AMR determinants. Clade III, formed from subclades IIIa and IIIb, was human dominated, and the most heterogeneous of the ST11 clades (median pairwise SNP difference 258.5, IQR=32.8–270). Clade IIIa consisted of two human and a poultry isolate, characterized by the presence of a mutation in the *pmrB* region, and clade IIIb consisted of two human isolates only.

Clade IV was also a relatively homogeneous clade (median SNP=33, IQR=31.2–39.8), consisting of poultry isolates detected near the northern border of the country (Fig. 1b) and one human isolate, all of which contained a point mutation in the *gyrA* region.

A small distinct population of two human and two poultry isolates, classified as ST183, formed clade V. The poultry isolates were both detected in the north of the country.

### Host distribution and evidence of transmission

The distribution of poultry and human isolates varied among clades; however, none of the differences were statistically significant (chi-square or Fisher’s exact test where any cell count was below 5, *P*>0.05, Table S3). At least one poultry isolate was present in every clade, except for subclade IIIb, which consisted of two human isolates that were genetically distinct from all poultry isolates (>250 SNPs).

In clades Ia, IIIa and IV, closely related genomes (<10 SNPs) were detected across both hosts. In the dominant clade Ia, human isolates MH_2, MH_4 and MH_9, collected in 2019, were genetically indistinguishable from Agri_122, a poultry isolate detected in 2020, and were highly related (≤5 SNPs) to a further three poultry isolates detected in 2019 and 2021. Within the same clade, four additional human isolates were highly related (≤5 SNPs) to poultry isolates collected across varying years (2019–2022).

Closely related isolates were also detected within the same host source (i.e. human–human and poultry–poultry pairs). In some instances, poultry isolates collected from the same local area, but in different years and on different farms, were highly related (≤5 SNPs) (e.g. three poultry isolates in clade II, Figs 1b and 2). Closely related poultry isolates were also found in geographically distant locations (e.g. indistinguishable isolates in clade Ia were recovered in the north and centre of the country, more than 150 km apart).

### Antimicrobial resistance

Overall, we found a very low prevalence of genetic AMR determinants (Table S2). All isolates contained the *aac(6′)-laa* gene, but no additional acquired AMR genes were detected. The majority of isolates (48/77) also did not contain any chromosomal point mutations. One or more mutations in the *gyrA* gene known to potentially confer resistance to quinolones (D87G, D87Y or S83F) were detected in 5/48 (10.4%) of the poultry and 2/29 (6.9%) of the human isolates. Furthermore, 14/48 poultry and 5/29 human samples contained a mutation *gyrA* Y100H, *parC N20S* or *parE* P302L; however, these have not been associated with clinical resistance. In addition, two human and one poultry isolates contained a mutation in the *pmrB* gene.

The presence of chromosomal point mutations was associated with clade, with the D87G mutation in the *gyrA* gene confined to clade IV isolates and the *pmrB* mutation to clade IIIa. The dominant clade Ia had a low prevalence of clinically significant mutations, with only 2/56 (3.6%) isolates carrying such mutations.

Phenotypic antimicrobial susceptibility testing of a representative selection of genotypes (*n*=8; Table S4) revealed that all isolates were pansusceptible, with the exception of those carrying *gyrA* mutation at position 87 (D87G or D87Y), which exhibited resistance to nalidixic acid and decreased susceptibility or resistance to ciprofloxacin (Table 1). One isolate carrying gyrA mutations at positions 83 and 253 was not tested.

**Table 1.** Genotype of *S.* Enteritidis isolates isolated in Israel (2019–2022) and the corresponding phenotype

No. of point mutations	Point mutation	Poultry	Human	Phenotype (n tested)
0	na	28	20	Pansusceptible (2)
1	*pmrB* G73D	1	2	Pansusceptible (1)
	*parC* N20S	1	0	Pansusceptible (1)
	*parE* P302L	11	5	Pansusceptible (1)
	*gyrA* D87G	4	1	Nalidixic acid – R, ciprofloxacin – D (1)
	*gyrA* D87Y	1	0	Nalidixic acid – R, ciprofloxacin – R (1)
	*gyrA* Y100H	2	0	Pansusceptible (1)
2	gyrA S83F, gyrA K253N	0	1	na

R=resistant (nalidixic acid (MIC ≥32 µg ml−1, ciprofloxacin MIC ≥1 µg ml−1).

D=decreased susceptibility to ciprofloxacin (MIC ≥0.12 and <1 µg ml−1).

na=not tested.

MIC, minimum inhibitory concentration.

### Virulence plasmid of *S*. Enteritidis

All except three ST11 isolates contained the plasmid replicons IncFIB(S) and IncFII(S); however, we did not detect a discrete plasmid in the hybrid assembly.

The isolates belonging to ST183 were missing the IncFIB plasmid replicon.

### Israeli isolates in the global context (analysis B)

Major branching of the phylogenetic tree placed the majority of the global isolates into two clades belonging to ST11, or closely related variants (Fig. 3). Three of the small Israeli clades and 12/115 (10.4%) of the global isolates sat outside these major clades.

**Fig. 3.**
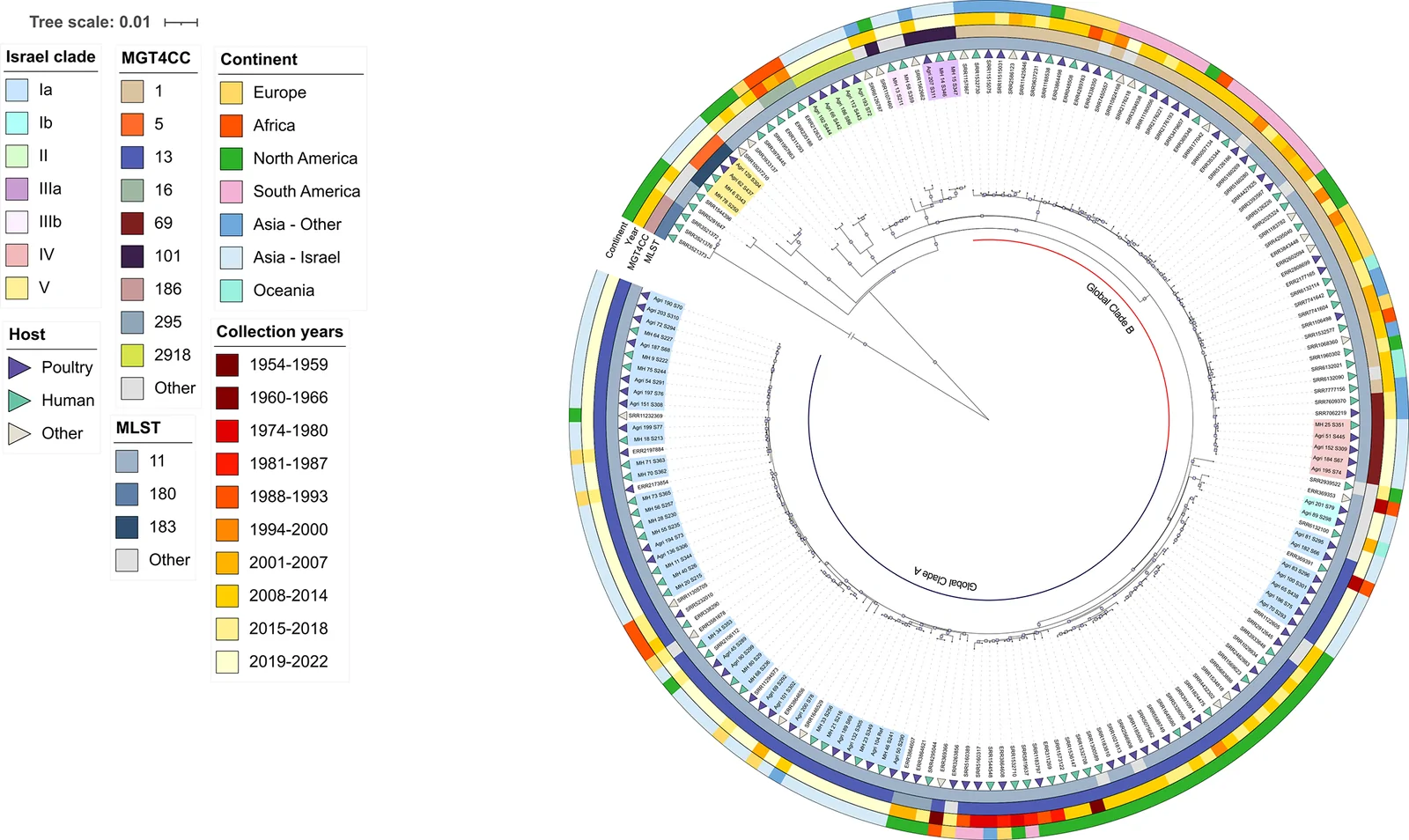
A maximum-likelihood phylogenetic tree of Israeli isolates (*n*=67) together with isolates representative of the global diversity of *S*. Enteritidis (*n*=115) downloaded from the NCBI was reconstructed. A hybrid assembly of *S*. Enteritidis (Agri_104) strain was used as a reference genome. A *Salmonella* Dublin strain (SRR1636692) was used to root the tree (not shown). Branch support for the trees was assessed using 1,000 bootstrap replicates. Bootstrap values of>80 are indicated (grey squares). The branch of outlier clade ST180 was truncated for improved visualization. The host, MLST type, MGT4CC type, year of collection and continent of origin of each isolate are displayed. Israeli clades are marked in colour blocks of the isolate names.

All isolates from the dominant Israeli clade I and 51/115 (44.3%) of global isolates, including the ten historical isolates collected between 1954 and 1987, formed the Global clade A. Isolates predominantly shared the same MGT4CC-13, and all continents were represented, including the majority of the North American isolates [31/47 (66.0%)].

Global clade B contained 52/115 (45.2%) of global isolates, mainly from the MGT4CC-1, which was the most prevalent MGT4CC type found globally. The MGT4CC-1 was not found in Israel, yet Israeli isolates from clade IV, assigned as MGT4CC-69, clustered together as part of Global clade B. The most closely related global isolates, also assigned to MGT4CC-69, were found in human and poultry hosts in Asia (specifically Lebanon and Iraq), in the years 2015–2018.

Israeli clades II, III and V were not included in the major global clades. The poultry-only Israeli clade II was assigned to a unique cluster complex (MGT4CC-2918), not shared with any of the global isolates. The human-dominated Israeli clade III was highly similar to three isolates detected from other sources (in these cases all food products) from Asia (specifically Bangladesh and Taiwan) and North America.

The Israeli clade V (ST183) and the ST180 isolates were the only non-ST11 clades. The ST180 isolates were found in North America, only in humans, and formed an outlier clade genetically distant from any other isolate (>20,000 SNP difference). The remaining global isolates were a genetically heterogeneous mix of human and non-poultry isolates, from a variety of years (1988–2018) and continents [North America (*n*=5), Africa (*n*=3) and Europe (*n*=1)].

## Discussion

*S*. Enteritidis isolates from layer hen flocks and human clinical cases in Israel during the period 2019–2022 were represented by five clades which belonged predominantly to the common ST11. In the global context, the Israeli dominant clade and one smaller clade were closely related to each of the two major lineages circulating globally. The remaining three smaller Israeli clades sat apart from the major lineages, highlighting the genetic diversity found within the country.

The overlap in genetic variation between human and poultry isolates in clade I is consistent with shared circulation. Notably, there were seven human isolates that were either indistinguishable from or highly related (≤5 SNPs) to poultry isolates in this clade, collected across multiple years (2019–2022), consistent with transmission. Although the direction of transmission cannot be established, given the broad exposure of humans via the consumption of eggs, poultry to human transmission is the more biologically plausible direction, and consistent with the broader literature [60, 61]. The persistence of these genetically similar isolates across the study years, and the geographical distribution over the country, suggests that this dominant clade is well established and is actively circulating in both host populations. Transmission dynamics within the poultry sector itself were also apparent: closely related strains (≤5 SNPs) were recovered from different farms in the same geographic area across multiple years, consistent with ongoing horizontal transmission between flocks. Considering flocks are culled and farms are disinfected after a positive finding of *S*. Enteritidis, possible vehicles for this transmission could be from external sources such as contaminated vehicles, feed [62, 63], rodents [64], arthropods [65], birds or wildlife [66, 67].

While clade I provides evidence for shared circulation, the smaller clades were dominated by either poultry (clades II and IV) or humans (clade III). Geographically, the smaller poultry clades were largely restricted to the northern region of the country, where the majority of farms are located (Fig. 1). Clade II was a poultry-only clade with no corresponding human isolates identified. In contrast, subclade IIIb consisted only of two human isolates that were genetically distant from all poultry isolates in the datasets (>250 SNPs). This finding may suggest an alternative non-poultry source of infection. The possibility of non-poultry reservoir hosts is further supported at the population level: despite extensive mitigation efforts in the Israeli poultry sector, including continuous surveillance, culling of positive flocks and farm disinfection, human *S*. Enteritidis incidence has remained persistently elevated [17], a pattern also observed in Europe, where no significant correlation has been reported between trends in human cases and the prevalence of *S*. Enteritidis in laying hen flocks over the past decade [2]. It should also be noted that Israeli isolates from chicken meat and other food-from-animal origin such as dairy, beef and pork were not included in this study, and these represent additional pathways that may contribute to human cases.

Israeli clade V, which contained both human and poultry isolates of ST183, is an interesting subpopulation distinct from the common ST11. This ST has been associated with human cases and wild animals such as hedgehogs in the UK [68] and Germany [69] and wild boar in Spain [26]. A recent study from New Zealand linked human cases to contact with farm animals [67], but this ST type has been rarely documented in poultry. The two ST183 poultry isolates were both detected in the northern part of the country, in localities ~30 km apart and adjacent to nature reserves. Despite sharing a clade, one human ST183 isolate was not closely related to the poultry ST183 sequences (>300 SNP difference), and therefore, we cannot infer direct transmission. Given the proximity to natural habitats, it is possible that wildlife, including hedgehogs or wild boar commonly found in northern Israel, or other non-poultry hosts, acted as reservoirs for this ST. However, in the absence of isolates from other species or the environment in the Israel cohort, these alternative pathways remain speculative. This novel finding nonetheless raises the possible role of non-poultry hosts which warrants further investigation.

The vast majority of Israeli *S*. Enteritidis isolates (90.9%) demonstrated a pansusceptible genotype, and no isolates were multidrug-resistant (i.e., simultaneously resistant to antibiotics from three or more drug classes). However, all but three of the ST11 isolates carried the plasmid replicons IncFIB(S) and IncFII(S), which are associated with the virulence plasmid commonly found in *S*. Enteritidis [70], and associated with the ability to produce systemic disease in humans [71]. All isolates carried the *aac(6′)-laa* gene; however, this gene is well established as cryptic and transcriptionally silent in *Salmonella* and is not considered to confer functional resistance [72]. Where additional genetic determinants were identified (9.1% of isolates), they were associated with fluoroquinolone resistance [73]. This is consistent with European surveillance data from 2021 and 2022 where multidrug resistance in *S*. Enteritidis from human isolates is classified as very low (2.4%), and resistance is highest to nalidixic acid (26%) and ciprofloxacin (22.8%) [8]. The presence of genetic determinants associated with fluoroquinolone resistance in isolates predominantly obtained from layer flocks in Israel is surprising, as fluoroquinolone use is not common in this sector. While the broiler sector commonly uses the beta-lactam amoxicillin, followed by fluoroquinolones, antimicrobial use is less common in the layer sector, and tetracyclines and macrolides are most commonly used (personal communication: Wiseman, A; EPB). These findings reinforce the importance of continued AMR surveillance in *S*. Enteritidis, particularly for fluoroquinolones and extended-spectrum cephalosporins, which remain the antimicrobial classes of choice for the treatment of invasive human salmonellosis [61]. Notably, all isolates in clade IV carried fluoroquinolone resistance determinants, and given their detection in both human and poultry hosts, this subpopulation warrants targeted ongoing surveillance.

*Salmonella* Enteritidis has been associated with a degree of intrinsic tolerance to colistin, with elevated minimum inhibitory concentrations relative to other serotypes in the absence of known genetic determinants [74]. Three isolates in this study carried a mutation in the *pmrB* gene, part of the *pmrCAB* operon involved in lipopolysaccharide modification and linked to colistin tolerance [75]. However, consistent with findings from other studies [8, 76], this mutation did not confer phenotypic resistance.

The global phylogenetic analysis placed the majority of isolates into two major lineages corresponding to the previously described ‘Atlantic’ (global clade A) and ‘Global’ (global clade B) lineages. Global clade A comprised predominantly of MGT4CC-13 isolates, including the dominant Israeli clade I, and contained the earliest isolates in our dataset, in keeping with the inference that this lineage originated from mid-twentieth century pandemics [7]. Isolates from this clade were broadly distributed across the globe, with a particular concentration of North American isolates. Global clade B was predominantly MGT4CC-1, a cluster complex recently identified as the predominant cluster complex in Australia [28] and China [11], and is also prevalent across North America and Europe according to the MGT database [27]. Israeli clade IV was assigned MGT4CC-69, clustered within the global clade B and was most closely related to isolates from Lebanon and Iraq, sharing the same cluster complex. Interestingly, a recent study from Lebanon [77] found *S*. Enteritidis to be the most prevalent serotype detected in humans and contaminated food products. Of the Lebanese isolates available on the MGT database, the majority of human (48/62) and poultry (11/11) are MGT4CC-69. Given the lack of commercial trade between Israel and Lebanon, this could represent a shared external source of breeding stock, or we could speculate that this is a subpopulation that is circulating in the border region, with reservoirs in wild animals or birds providing a plausible (but unverified) transmission pathway.

The three remaining Israeli clades II, III and V were situated outside the two major global lineages. Israeli clade II was assigned a unique cluster complex (MGT4CC-2918) not shared with any global isolate in the dataset, while clade III showed closest similarity to isolates recovered from food products in Bangladesh, Taiwan and North America, though the epidemiological significance of this association remains unclear.

The absence of MGT4CC-1, the predominant cluster complex in our global dataset, from both Israeli human and poultry isolates was unexpected, especially given Israel’s geographic position at the junction of Europe, Asia and Africa and the high rate of international travel among its citizens. Additionally, introduction of strains is possible via the importation of table eggs and poultry breeding stock; however, these pathways are subject to EU regulations that include strict *Salmonella* control [78], and as previously mentioned, *S*. Enteritidis was not detected in any layer breeder flocks during routine surveillance across the study period, suggesting that this is unlikely to be a major transmission route. It is possible, however, that the absence of this predominant global complex cluster may be a result of a low prevalence rather than a genuine absence.

A limitation of this study is the small size of the human clinical dataset and the lack of patient metadata and epidemiological investigations. We acknowledge that the use of isolates from passive surveillance of clinical cases will represent a bias towards strains capable of causing symptomatic disease in humans and is not necessarily representative of all circulating lineages. In addition, our data provides a snapshot in time (of the 4-year study period) and may not fully capture the genetic diversity that has been circulating in poultry and clinical settings.

This study represents the first genomic characterization of *S*. Enteritidis in Israel, integrating isolates from commercial layer hen flocks and human clinical cases within a global phylogenetic framework. These results provide a baseline for ongoing surveillance of *S*. Enteritidis in Israel and highlight the importance of integrated One Health monitoring. Future studies incorporating larger, metadata-linked human clinical datasets, environmental sampling and surveillance of wildlife and non-poultry hosts will be essential to fully elucidate the transmission dynamics of this pathogen and inform targeted public health interventions.

## Supplementary material

10.1099/mgen.0.001878 Supplementary Material 1.

## Abbreviations

AMR: antimicrobial resistance
CLSI: Clinical and Laboratory Standards Institute
EPB: Egg and Poultry Board
IQR: interquartile range
MGT: multilevel genome typing
MLST: Multilocus Sequence Type
MOH: Ministry of Health
NCBI: National Center for Biotechnology Information
NTS: non-typhoidal *Salmonella*
ST: sequence type
WGS: whole-genome sequencing
